# MicroRNAs Engage in Complex Circuits Regulating Adult Neurogenesis

**DOI:** 10.3389/fnins.2018.00707

**Published:** 2018-11-05

**Authors:** Laura Stappert, Frederike Klaus, Oliver Brüstle

**Affiliations:** Institute of Reconstructive Neurobiology, Life & Brain Center, University of Bonn Medical Center, Bonn, Germany

**Keywords:** microRNAs, adult neurogenesis, hippocampus, neural stem cells, neurons, feedback loops, miRNA convergence

## Abstract

The finding that the adult mammalian brain is still capable of producing neurons has ignited a new field of research aiming to identify the molecular mechanisms regulating adult neurogenesis. An improved understanding of these mechanisms could lead to the development of novel approaches to delay cognitive decline and facilitate neuroregeneration in the adult human brain. Accumulating evidence suggest microRNAs (miRNAs), which represent a class of post-transcriptional gene expression regulators, as crucial part of the gene regulatory networks governing adult neurogenesis. This review attempts to illustrate how miRNAs modulate key processes in the adult neurogenic niche by interacting with each other and with transcriptional regulators. We discuss the function of miRNAs in adult neurogenesis following the life-journey of an adult-born neuron from the adult neural stem cell (NSCs) compartment to its final target site. We first survey how miRNAs control the initial step of adult neurogenesis, that is the transition of quiescent to activated proliferative adult NSCs, and then go on to discuss the role of miRNAs to regulate neuronal differentiation, survival, and functional integration of the newborn neurons. In this context, we highlight miRNAs that converge on functionally related targets or act within cross talking gene regulatory networks. The cooperative manner of miRNA action and the broad target repertoire of each individual miRNA could make the miRNA system a promising tool to gain control on adult NSCs in the context of therapeutic approaches.

## Introduction

**FIGURE 1:**
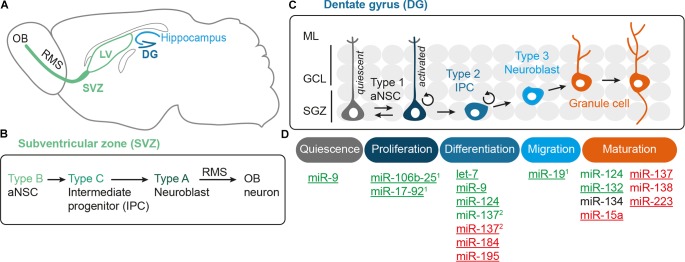
MicroRNAs as key regulators of the multistep process of adult neurogenesis. **(A)** The adult mouse brain contains two main neurogenic niches, i.e., the subventricular zone (SVZ) of the lateral ventricle (LV), which produces neurons for the olfactory bulb (OB), and the subgranular zone (SGZ) of the hippocampal dentate gyrus (DG). **(B)** The adult neural stem cells (aNSCs) of the subventricular zone are called type B cells. They give rise to intermediate progenitors (IPCs, type C cells), which after a few cell cycles develop into type A neuroblasts that migrate along the rostral migratory stream (RMS) to the OB before terminal differentiation (Doetsch et al., 1997). **(C)** The aNSCs of the SGZ of the dentate gyrus are called type 1 cells and the IPCs to which they develop are designated as type 2, the neuroblasts as type 3 cells (Seri et al., 2004). The neuroblasts generated from the SGZ migrate up into the granule cell layer (GCL), where they maturate into granule cells, extend dendrites into the molecular layer (ML), and grow axons. **(D)** Adult neurogenesis is a multistep process that begins with activation of quiescent aNSCs to re-enter cell cycling, followed by a proliferation phase of aNSCs, which eventually develop into IPCs and further differentiate into neuroblasts. The last steps of adult neurogenesis involve neuroblast migration and functional maturation into terminally differentiated neurons. For all these steps, regulatory miRNAs have been identified that either have a positive (green), negative (red), or modulatory (black) effect. Underlined miRNAs have been studied in the context of adult neurogenesis, whereas the function of the other miRNAs has been inferred from studies addressing developmental neurogenesis. The miRNAs miR-106b-25^(1)^, miR-17-92^(1)^ and miR-19^(1)^ belong to the family of polycistronic miR-17 clusters. There are divergent results for miRNA-137^(2)^, which was found to have a pro-differentiation effect in embryonic NSCs (Sun et al., 2011), but an anti-differentiation effect in adult NSCs (Szulwach et al., 2010).

Neural stem cells (NSCs) are self-renewing, multipotent progenitors that generate all neurons and glial cells of the mammalian central nervous system (CNS). In the adult mammalian brain, a limited number of adult neural stem cells (aNSCs) persists in the subventricular zone (SVZ) of the lateral ventricles and the subgranular zone (SGZ) in the hippocampal dentate gyrus as the two main neurogenic niches (Figures 1A–C). The composition and functionality of these germinal zones has been most extensively studied in rodents (reviewed by Kempermann et al., 2015; Pino et al., 2017). Similar regions containing neurogenic progenitor cells have been described in the SVZ and dentate gyrus of the adult human brain (Eriksson et al., 1998; Roy et al., 2000; Nunes et al., 2003). Neurogenesis within the human SVZ declines during infancy (Sanai et al., 2011), whereas several studies have pointed to a quite substantial generation of dentate granule neurons in humansthroughout life (Spalding et al., 2013; Boldrini et al., 2018). This view has been challenged by a recent report suggesting that hippocampal neurogenesis decreases dramatically after the first years of life in both humans and macaques [Sorrells et al. (2018); see also Kempermann et al. (2018) for a statement regarding the recent discussion on the relevance of adult hippocampal neurogenesis in humans]. Nevertheless, there is a strong interest in understanding the mechanisms regulating adult neurogenesis (reviewed by Kempermann et al., 2015; Peng and Bonaguidi, 2018). Research in this regard is motivated by the fact that the hippocampus is involved in memory and learning, which led to the hypothesis that adult neurogenesis could play an important role in cognition (reviewed by Gonçalves et al., 2016). Furthermore, a series of studies revealed that adult hippocampal neurogenesis in rodents can be modulated by experiential and environmental conditions as well as by aging (reviewed by Toda and Gage, 2018). Altered hippocampal neurogenesis has been linked to a number of pathological conditions, such as ischemia- or epilepsy-induced insults, mood disorders, and neurodegenerative diseases (reviewed by Pino et al., 2017).

**FIGURE 2:**
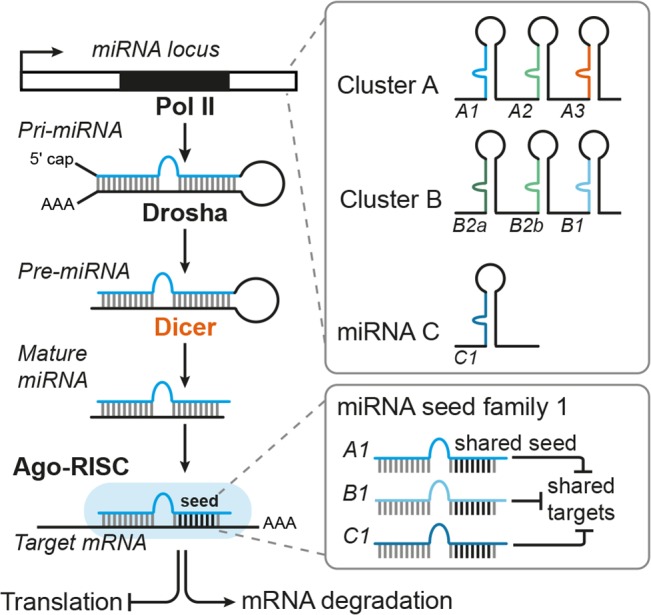
Canonical miRNA biogenesis and function. miRNA genes are transcribed by RNA Polymerase II (Pol II) and then processed by the sequential action of Drosha and Dicer to liberate the mature miRNA duplex, which is then loaded onto Argonaut proteins (Ago) to form the RNA-induced silencing complex (RISC). As part of the RISC, miRNAs can bind to target mRNAs whereby binding of the miRNA seed sequence is the key requirement for mRNA target recognition. Thus, miRNAs with the same seed sequence are thought to target overlapping sets of mRNAs and are assigned to the same miRNA seed family. miRNA genes can be either monocistronic (miRNA C) or polycistronic (clusters A and B) containing several miRNAs that are usually expressed as one pri-miRNA transcript. Some polycistronic clusters comprise miRNA homologs that have emerged via duplication events (cluster B with miR-B2a, B2b as homologous miRNAs) whereas other clusters consist of miRNAs belonging to different seed families (here exemplarily designated as seed family 1 (in blue) with miRNAs A1, B1, C1, and seed family 2 (in green) with miRNAs A2, B2a, and B2b). For more details on miRNA biogenesis and polycistronic miRNA clusters, see the reviews from Ha and Kim (2014) and Olive et al. (2015), respectively.

Adult neurogenesis involves multiple steps that have to be tightly regulated, i.e., aNSC activation, proliferation, differentiation into neural progeny as well as survival, migration, and functional maturation of the adult-born neurons (Figure 1D). Recent evidence indicate that microRNAs (miRNAs) can be placed in midst of the regulatory mechanisms operating in adult neurogenesis (reviewed by Lopez-Ramirez and Nicoli, 2014; Murao et al., 2016; Encinas and Fitzsimons, 2017). miRNAs are short (22 nucleotide long) single-stranded RNA molecules that post-transcriptionally repress gene expression by complementary binding to mRNA targets (reviewed by Bartel, 2009). miRNA genes are transcribed as hairpin-shaped transcripts, called pri-miRNAs, which are sequentially processed to liberate the mature miRNAs (Figure 2). Some pri-miRNAs are polycistronic and encode for several mature miRNAs (reviewed by Olive et al., 2015). The final and essential cleavage step of miRNA biogenesis is carried out by the ribonuclease Dicer, and genetic ablation studies for Dicer have been used to assess the overall importance of the miRNA system. Mature miRNAs are then loaded onto Argonaut (Ago) proteins to form the RNA-induced silencing complex (RISC) through which they target mRNAs for translational inhibition or mRNA degradation. miRNAs often come as families whose members share a common seed sequence and are thought to have similar functions (Figure 2) (reviewed by Ha and Kim, 2014). During brain development, miRNAs regulate neural progenitor proliferation, neurogenic and gliogenic differentiation as well as maturation and functional integration of neurons (reviewed by Bian et al., 2013; Barca-Mayo and De Pietri Tonelli, 2014; Stappert et al., 2014). Likewise, miRNAs play important roles in regulating cell fate decisions in the adult SVZ and SGZ (reviewed by Lopez-Ramirez and Nicoli, 2014; Murao et al., 2016) and have been linked to diseases associated with these compartments, e.g., epilepsy (Bielefeld et al., 2017), stroke (Khoshnam et al., 2017), and neurodegenerative disorders (Qiu et al., 2014).

In many cases, miRNAs act in concert with transcription factors and chromatin modifiers to control gene expression in NSCs (of embryonic or adult origin), thereby affecting NSC number and their ability to generate differentiated progeny.

In this review, we discuss the physiological role of miRNAs during adult neurogenesis following the route from aNSC maintenance to neuronal differentiation and maturation of newborn neurons. Given its discussed relevance for human neurogenesis and cognition, we will mainly focus on hippocampal neurogenesis. However, in some cases we will also refer to data generated in other compartments, e.g., the adult SVZ or the developing brain, to highlight important principles of miRNA function during neurogenesis (see Figure 1D and Tables 1, 2 for an overview of the functions of the miRNAs presented here). In the first part, we depict how miRNAs control the balance between quiescent and activated aNSCs contributing to homeostasis and plasticity in response to neurogenic stimuli. In the second part, we describe how miRNAs influence the neurogenic output of the aNSC niche by modulating neuronal differentiation, survival, and functional integration. In this context, we delineate the interactions of miRNAs with gene regulatory networks controlling adult neurogenesis and focus on miRNAs that converge by targeting functionally associated genes. We end by summarizing the diverse roles of miRNAs during adult neurogenesis and discuss the importance of target multiplicity and miRNA cooperativity as key features of the miRNA system. Finally, we speculate how miRNAs may contribute to aNSC heterogeneity and give an outlook on how knowledge on miRNA-based regulation could be further increased and eventually exploited to facilitate neuroregeneration in the adult human brain.

**Table 1:** MicroRNAs modulating proliferation and differentiation during adult neurogenesis.

MicroRNA	Main model system	Observed main effect	miRNA regulator	Target mRNA	Reference
**let-7b**	Mouse primary neonatal forebrain NSCs	let-7 decreases NSC proliferation		*Hmga2*	Nishino et al., 2008
	Mouse embryonic VZ, **primary adult forebrain NSCs**	**let-7 promotes neuronal differentiation**		***Tlx, CyclinD1 (Ccnd1)***	Zhao et al., 2010
**miR-17-92 cluster**	**Nestin-CreER miR-17-92 KO/OE mice, adult hippocampus**	**miR-17-92 promotes aNSC proliferation and rescues stress-induced impairment of neurogenesis**		***Sgk1***	Jin et al., 2016
**miR-25 (miR-106b-25) cluster**	**Mouse primary adult forebrain NSCs**	**miR-25 promotes aNSC proliferation**	**FOXO3**	***Foxo3* (predicted)**	Brett et al., 2011
**miR-9**	Mouse embryonic VZ, **primary adult forebrain NSCs**	**miR-9 promotes neuronal differentiation**	**TLX**	***Tlx***	Zhao et al., 2009
	Human embryonal carcinoma cell line	miR-9/9^∗^ and the REST silencing complex form a double negative feedback loop	REST	*Rest, CoRest (Rcor2)*	Packer et al., 2008
	Human neuroblastoma cell line	miR-9 expression is inhibited by Rest in undifferentiated cells and promoted by CREB in differentiated cells, miR-9 targets *Rest* forming a feedback loop	REST, CREB	*Rest*	Laneve et al., 2010
	**Mouse primary neonatal NSCs, adult SVZ**	**miR-9 OE promotes neuronal differentiation; miR-9 decreases Notch signaling dependent on FOXO1**	**FOXO1 (predicted)**	***Foxo1***	Kim et al., 2015
	**Adult zebrafish brain**	**Nuclear localized non-canonical miR-9 maintains aNSC quiescence**	**TNRC6 (for nuclear shuttling)**	**Notch signaling (indirect positive effect)**	Katz et al., 2016
**miR-124**	Mouse embryonal carcinoma cell line	REST prevents miR-124 expression in neural progenitors	REST		Conaco et al., 2006
	Chick neural tube, mouse embryonal carcinoma cell line	miR-124 promotes neuronal differentiation	REST	*Scp1*	Visvanathan et al., 2007
	**Mouse adult SVZ: injection of miR-124 OE retrovirus, infusion of miR-124 inhibitor**	**miR-124 promotes neuronal differentiation and is necessary for SVZ regeneration**		***Sox9***	Cheng et al., 2009
	**Mouse adult SVZ: injection of miR-124 OE/sponge lentivirus**	**miR-124 OE leads to precocious neuronal differentiation and aNSC exhaustion; miR-124 inhibition represses neuronal differentiation and promotes glial differentiation**			Akerblom et al., 2012
**miR-137**	**Mouse primary adult forebrain NSCs, DG retroviral injection**	**miR-137 OE promotes aNSC proliferation and represses neuronal differentiation**	**MECP2**	***Ezh2***	Szulwach et al., 2010
	Mouse embryonic NSCs, *in utero* electroporation into the lateral ventricle	miR-137 OE inhibits NSC proliferation and accelerates neuronal differentiation	TLX (via LSD1)	*Lsd1*	Sun et al., 2011
**miR-184**	**Mouse primary adult DG NSCs, DG retroviral injection**	**miR-184 promotes aNSC proliferation**	**MBD1**	***Numbl***	Liu et al., 2010
**miR-195**	**Mouse primary adult DG NSCs, DG retroviral injection**	**miR-195 promotes aNSC proliferation**	**MBD1**	***Mbd1***	Liu et al., 2013


*Overview of miRNAs (in ascending numerical order) discussed in this review that modulate proliferation and differentiation of (adult) neural progenitor cells, their upstream regulators, target mRNAs, and the model systems they have been studied in. Findings relating to adult neurogenesis are highlighted in bold. (a) NSC, (adult) neural stem cells; DG, dentate gyrus; KO, knockout; OE, overexpression; SVZ, subventricular zone; VZ, ventricular zone.*

**Table 2:** Putative impact of miRNAs on neuronal migration and neuronal morphogenesis in the context of adult neurogenesis.

MicroRNA	Main model system	Observed main effect	miRNA regulator	Target mRNA	Reference
**miR-15a**	**Floxed *Mecp2* mice, DG retroviral grafting of Cre-GFP/miR-15a sponge,** primary neonatal cortical/hippocampal neurons	**miR-15a impairs dendrite maturation, miR-15a inhibition rescues *Mecp2*-deficiency-induced neuronal maturation deficits**	**MECP2**	***Bdnf***	Gao et al., 2015
**miR-19**	**Mouse primary adult DG NSCs, DG and SVZ retroviral injection**	**miR-19 promotes migration of neurons**		***Rapgef2***	Han et al., 2016
**miR-124**	Rat primary embryonic hippocampal neurons	miR-124 increases axonal and dendrite complexity		*Rhog*	Franke et al., 2012
**miR-132**	Rat primary neonatal cortical neurons	CREB induces miR-132 expression downstream of BDNF, miR-132 promotes neurite outgrowth	CREB	p250Gap (*Arhgap32)*	Vo et al., 2005
	Rat primary neonatal cortical neurons	miR-132 and MECP2 form a feedback mechanism via BDNF	BDNF via CREB	*Mecp2*	Klein et al., 2007
	Rat primary neonatal hippocampal neurons	miR-132 promotes dendrite growth and spine maturation		p250Gap (*Arhgap32)*	Wayman et al., 2008
	**Floxed miR-213/132 mice and GFP-Cre DG retroviral injection**	**Deletion of miR-132 decreases dendrite length and arborization**			Magill et al., 2010
**miR-134**	Rat primary embryonic cortical/hippocampal neurons	miR-134 decreases size of dendritic spines	BDNF (indirect effect)	*Limk1*	Schratt et al., 2006
	***Sirt1* KO/Nestin-Cre mice, adult hippocampal lentiviral injection**	**miR-134 knockdown rescues *Sirt1*-deficiency-induced LTP and memory defects**	**SIRT1**	***Creb***	Gao et al., 2010
	*Mecp2* KO/OE mice, mouse primary embryonic cortical neurons	*Mecp2* OE inhibits dendrite growth and pri-miR-134 processing; miR-134 OE rescues dendrite growth defect	MECP2		Cheng T.-L. et al., 2014
**miR-134 part of miR-379-410 cluster**	Rat primary embryonic cortical/hippocampal neurons	miR-379-410 expression is regulated by neuronal activity via MEF2; miR-134 promotes dendrite outgrowth	MEF2	*Pum2*	Fiore et al., 2009
**miR-137**	**Mouse DG retroviral injection,** primary embryonic hippocampal neurons	**miR-137 OE inhibits dendrite morphogenesis**		***Mib1***	Smrt et al., 2010
**miR-138**	Rat primary embryonic cortical/hippocampal neurons	miR-138 decreases size of dendritic spines		*Apt1*	Siegel et al., 2009
**miR-223**	**Retroviral injection of miR-223 sponge into mouse DG,** miR-223 OE in human fetal NPCs	**miR-223 inhibits dendrite outgrowth**			Harraz et al., 2014


*List of the miRNAs (in ascending numerical order) and their respective up-stream regulators and target mRNAs associated with regulating neuron migration and morphogenesis during neurogenesis. Findings directly related to adult neurogenesis are highlighted in bold. DG, dentate gyrus; KO, knockout; LTP, long-term potentiation; NSC, neural stem cells; OE, overexpression; SVZ, subventricular zone.*

### MicroRNAs Regulating Activation and Proliferation of Adult Neural Stem Cells

Adult NSCs reside in a specialized microenvironment, the stem cell niche, which is composed of different cell types and extracellular matrix molecules and provides extracellular signals to regulate NSC homeostasis and differentiation (reviewed by Llorens-Bobadilla and Martin-Villalba, 2017). In the currently prevalent view, aNSCs are slowly dividing radial glia-like precursor cells that express *Nestin*, *Gfap*, as well as *Sox2* and are in close contact with the vasculature. The radial glia-like precursor cells of the dentate gyrus SGZ are further characterized by basal processes that span the granule cell layer. The radial glia-like NSCs (SVZ: type B cells, SGZ: type 1 cells) generate fast-dividing committed intermediate progenitors (IPCs, SVZ: type C cells, SGZ: type 2 cells), which then give rise to neuroblasts (SVZ: type A cells, SGZ: type 3 cells) (Doetsch et al., 1997; Seri et al., 2004; and reviewed by Pino et al., 2017) (see Figures 1B,C for an overview of the lineage relationships and nomenclature in the SVZ and SGZ). The SVZ neuroblasts migrate along the rostral migratory stream to the olfactory bulb where they differentiate into olfactory interneurons. The dentate gyrus neuroblasts generated from the SGZ move up into the granule cell layer before they differentiate into mature granule neurons (Figures 1A–C) (reviewed by Pino et al., 2017).

The majority of aNSCs is quiescent, which seems to be important to ensure long-term homeostasis of the niche throughout the life span of the organism. However, a small fraction of aNSCs has been shown to proliferate quite rapidly, and it has been suggested that aNSCs transition between activated proliferative and quiescent states (Figure 1C; Lugert et al., 2010). The number of aNSCs shuttling between these two states is modulated by neurogenic stimuli and during aging (van Praag et al., 1999; Lugert et al., 2010; Encinas et al., 2011). In fact, neurogenic activity seems to decline with age, whereby it is still unresolved whether this is due to an exhaustion of aNSCs, which once activated eventually lose their self-renewal capacity and terminally differentiate (Encinas et al., 2011; Ziebell et al., 2018), or an increased quiescence of the aNSCs pool (Lugert et al., 2010; and reviewed by Giachino and Taylor, 2015).

#### MicroRNAs Controlling the Balance Between Quiescent and Activated Proliferative aNSCs

There are many factors known to control the proliferative capacity of aNSCs (reviewed by Beckervordersandforth et al., 2015) including transcription factors and signaling pathways, such as Notch, BMP, Wnt, and insulin/IGF signaling. While for many of the players involved regulatory miRNAs have been identified, we will focus on such miRNA–target interactions that have been explicitly studied in the context of adult neurogenesis.

One pathway that is regulated by many miRNAs and plays a key role in controlling aNSCs proliferation is Notch signaling (reviewed by Giachino and Taylor, 2015). High Notch levels protect aNSC from activation as shown by studies in mouse SVZ (Kawaguchi et al., 2013) and SGZ (Semerci et al., 2017) as well as in zebrafish aNSCs (Chapouton et al., 2010). While studying adult neurogenesis in the zebrafish brain, which exhibits a widespread neurogenic capacity and contains regions similar to the rodent SVZ and SGZ, Katz et al. (2016) found that miR-9 potentiates Notch signaling to maintain aNSC quiescence. This function of miR-9 stands in apparent contrast to its previously described pro-differentiation effect in embryonic (reviewed by Roese-Koerner et al., 2017) and adult NSCs (Zhao et al., 2009; Kim et al., 2015) (see also Table 1). The proposed role of miR-9 during aNSC quiescence in zebrafish may, however, reflect a non-canonical function of miR-9 since Katz et al. (2016) found that miR-9 is actively transported to the nucleus by binding of miR-9-Ago complexes to the shuttle protein TNRC6 and that this nuclear concentration is critical for aNSC quiescence. Interestingly, nuclear localization of miR-9 was also detected in a subset of cells in the SVZ and SGZ neurogenic niche in the mouse brain, suggesting that the role of miR-9 in regulating aNSC quiescence might be conserved (Katz et al., 2016). Furthermore, nuclear concentration of miR-9 was also found to be increased in older versus younger mice, pointing to an age-dependent shift of the subcellular localization of miR-9 (Katz et al., 2016).

Another example of a miRNA-target pair implicated in aNSC maintenance is let-7 and *Hmga2*. miRNA let-7 and its target gene *Hmga2* show an inverse expression pattern in the murine SVZ during aging (Nishino et al., 2008). HMGA2 is a member of the high mobility group protein family that modulates gene expression as part of the enhanceosome (reviewed by Pfannkuche et al., 2009). Nishino et al. (2008) showed that HMGA2 promotes the proliferative capacity of fetal and adult NSCs by repressing expression of *Ink4a/Arf* locus, which encodes for the cell cycle inhibitors p16 (*Cdkn2a*) and p19 (*Cdkn2d*). However, HMGA2 seems not to bind to the *Ink4a*/*Arf* locus and it is not yet clear how HMGA2 represses expression of *Ink4*/*Arf*. Expression of *Hmga2* declines during aging, and premature loss of *Hmga2* impairs self-renewal in *in vitro* cultured forebrain aNSCs, which may contain both SVZ and SGZ aNSCs. Nishino et al. (2008) further found that *Hmga2* expression is regulated by let-7, whose expression increases with age. Furthermore, overexpression of let-7 decreases the proliferative capacity of *in vitro* cultured forebrain aNSCs mimicking the effect of *Hmga2* loss. Thus, the interaction of HMGA2 and let-7 may couple aNSC self-renewal and aging.

Quiescence of aNSCs is also regulated by FOXO3, which was initially identified as an age- and longevity-associated factor downstream of insulin/IGF signaling (reviewed by Martins et al., 2015). FOXO3 is necessary to maintain quiescent aNSCs in both the SVZ and SGZ (Paik et al., 2009; Renault et al., 2009) and was suggested to interact with the polycistronic miR-106b-25 cluster. This cluster is encoded in an intronic region of the protein-coding *Mcm7* gene, which is one of the FOXO3 target genes (Brett et al., 2011). In turn, miR-25, generated by the miR-106b-25 cluster, is critical for the proliferative capacity of *in vitro* cultured forebrain aNSC and is predicted to target *Foxo3* mRNA. Thus, miR-25 and FOXO3 might form a feedback loop regulating the self-renewal capacity of aNSCs (Brett et al., 2011). Unfortunately, the impact of miR-25 on *Foxo3* has not yet been experimentally validated. Nevertheless, an interaction between IGF signaling, FOXO3, and miR-25 to regulate NSC proliferation seems an attractive scenario, which might even be evolutionary conserved as the *C. elegans* ortholog of miR-25, cel-miR-253, has been shown to couple proliferation of blast cells to the nutritional state downstream of IGF signaling (Kasuga et al., 2013). Interestingly, ectopic expression of miR-25 can even re-instate cell cycling of post-mitotic zebrafish neurons (Rodriguez-Aznar et al., 2013). The relevant target gene in this context is p57 (*Cdkn1c*), which has been shown to pace adult hippocampal neurogenesis in mice by controlling aNSCs quiescence (Furutachi et al., 2013).

Taken together, these findings indicate the importance of miRNAs in regulating activation and proliferation of aNSCs, which is key for long-term homeostasis of the adult neurogenic niche.

#### MicroRNAs Regulating aNSC Activation Downstream of Neurogenic Modulators

Hippocampal neurogenesis is modulated by several physiological stimuli, such as physical exercise, environmental conditions, learning, and aging. Physical activation, for instance, has been shown to promote neurogenesis by inducing cell cycle entry of quiescent aNSCs (Lugert et al., 2010), while exposing mice to enriched environment enhances the survival of new neurons (van Praag et al., 1999). Interestingly, it has been demonstrated that exposure of mice to an enriched environment also leads to altered miRNA expression profiles in the hippocampus (Barak et al., 2013). In total, 29% of the miRNAs, including miR-124, were found to be down-regulated in response to enriched environment, while 8% (including miR-132 as the most increased miRNA) were up-regulated (Barak et al., 2013). Many of the differentially expressed miRNAs also showed an inverse differential expression in a mouse model for Alzheimer’s disease, which has been associated with impaired adult hippocampal neurogenesis. These data point to an important role of miRNAs in conferring plasticity of hippocampal neurogenesis in response to modulatory signals.

In further support of this, the polycistronic miR-17-92 cluster, which shares miRNA seed family members with the miR-106b-25 cluster, was recently shown to regulate hippocampal neurogenesis in a mouse model of chronic stress (Jin et al., 2016). Stress and mood-related disorders have adverse effects on the neurogenic activity of the hippocampus, while antidepressant treatment may reverse this impairment. In fact, mood and psychiatric disorders have been associated with dysfunctional hippocampal neurogenesis (reviewed by Mahar et al., 2014). Mouse transgenic gain- and loss-of-function models revealed that miR-17-92 is required for aNSC proliferation in the dentate gyrus and that alterations of miR-17-92 expression have a strong impact on hippocampal neurogenesis and evoke changes in stress and anxiety-related behavioral tests. Mice depleted for miR-17-92 showed a reduced hippocampal neurogenic activity and exhibited anxiety-like behavior. It was further shown that miR-17-92 targets *Sgk1*, a downstream effector of glucocorticoid receptor signaling involved in cellular stress response, and that miR-17-92 can rescue the impairment of neural progenitor proliferation induced by corticosterone treatment (Jin et al., 2016). Expression of miR-17-92 itself is down-regulated upon chronic stress suggesting miR-17-92 as a physiological effector of stress-impaired hippocampal neurogenesis. Of note, miR-17-92 expression in aNSCs might change during aging as indicated by a recent study reporting a reduced miR-17-92 expression in neurogenic niches of old versus young *N. furzeri* fish (Terzibasi Tozzini et al., 2014).

### MicroRNAs Modulate Adult Neuronal Differentiation by Acting in Concert With Gene Expression Regulators

Activated aNSCs give rise to IPCs, which then differentiate into neuroblasts that further mature into the respective neurons, i.e., olfactory bulb neurons (generated from the SVZ) and dentate granule cells (generated from the SGZ) (Figure 1). miRNAs have been shown to regulate the transition from proliferation to neuronal differentiation both during embryonic and adult neurogenesis. In this context, miRNAs are often found to interact with gene expression regulators forming feedback loops as we delineate in the following paragraphs. In fact, a recurrent feature of the miRNA’s mode of action is that several miRNAs act on the same transcriptional regulator, which in turn modulates the expression of its regulatory miRNAs, thereby forming feedback loops to fine-tune gene expression (reviewed by Arora et al., 2013; Lopez-Ramirez and Nicoli, 2014; Osella et al., 2014; Murao et al., 2016).

#### MicroRNA-124 and miR-9 Are Part of a Gene Regulatory Network Controlling Developmental and Adult Neurogenesis

One of the most studied miRNAs expressed in the brain are miR-124 and miR-9, which are not only able to induce neuronal differentiation of embryonic NSCs (reviewed by, e.g., Akerblom and Jakobsson, 2014; Roese-Koerner et al., 2013) but can even instruct a neurogenic gene expression program in non-neuronal cells (Yoo et al., 2011). Both miR-124 and miR-9 are widely expressed in the mouse adult brain including the hippocampus (Bak et al., 2008) and play important roles during adult neurogenesis. miRNA-124 has been shown to promote neuronal differentiation in the mouse SVZ by targeting the transcription factor SOX9 (Cheng et al., 2009). Stable overexpression of miR-124 in the mouse SVZ initially boosts neuronal differentiation but ultimately leads to premature exhaustion of the aNSC pool and loss of neurogenic activity (Akerblom et al., 2012). miRNA-9 is also expressed in the adult mouse SVZ, where it promotes neuronal differentiation (Zhao et al., 2009; Kim et al., 2015). One of the *bona fide* miR-9 targets identified in this context is *Foxo1*, which is related to *Foxo3*, and was shown to maintain aNSC self-renewal by acting in concert with Notch signaling (Kim et al., 2015). These findings are in contrast to the suggested role of miR-9 in maintaining quiescence in the zebrafish neurogenic niche (Katz et al., 2016), and it is not clear whether miR-9 impacts on both aNSC quiescence and differentiation in the adult mouse brain.

**FIGURE 3:**
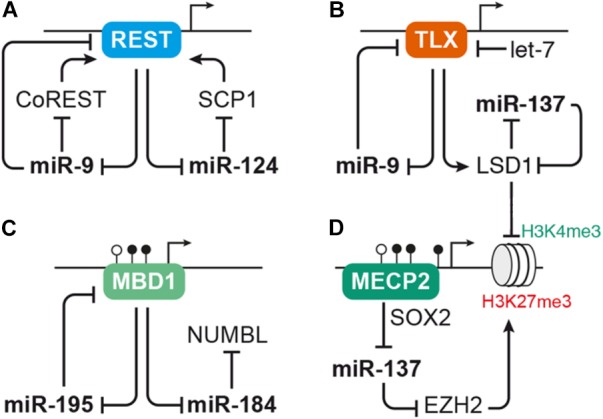
MicroRNAs interact with transcription factors and epigenetic regulators to control aNSC proliferation and differentiation. **(A)** The transcriptional repressor REST serves as regulatory hub for several miRNAs, including pro-neural miR-9 and miR-124. REST represses expression of miR-9 and miR-124, which in turn interfere with the activity of the Rest complex by targeting *Rest* or its cofactors *CoRest* and *Scp1*. **(B)** Another transcription factor interacting with miRNAs in the context of NSC self-renewal and differentiation is TLX. TLX has a direct repressive effect on miR-9 expression and also inhibits expression of miR-137 via recruiting the histone demethylase LSD1. The activity of TLX itself is regulated by miR-9 as well as let-7, which both target *Tlx*, and miR-137, which targets *Lsd1*. **(C–D)** miRNAs often interact with epigenetic regulators, such as mediators of DNA methylation and histone modifiers. **(C)** Expression of miR-184 and miR-195 is suppressed by binding of the methyl-CpG-binding protein MBD1 to their genomic loci. miRNA-195, in turn, binds to *Mbd1* mRNA forming a negative feedback loop, while miR-184 targets *Numbl*. **(D)** MECP2 cooperates with the neural progenitor transcription factor SOX2 to repress miR-137 expression. miRNA-137 may regulate global gene transcription through repressing the expression of histone modifiers, i.e., EZH2, which induces repressive H3K27 trimethylation (me3) marks, and LSD1, which erases activating H3K4me3 marks. LSD1 and MECP2 are also recruited by the REST/CoREST complex, thus forming another node through which the miRNAs discussed here may cross talk with each other. For sake of simplicity, these interactions were not depicted here. For a detailed review on the cross talk between miRNAs and epigenetic regulators, see Jobe et al. (2012).

miRNA-124 and miR-9 have been shown to interact with an overlapping set of gene expression regulators that play important roles during NSC differentiation (reviewed by Stappert et al., 2014). Several of the transcription factors interacting with miR-124 and miR-9, e.g., REST and TLX, are also relevant in the context of adult neurogenesis. We will center our discussion on the miRNA-based circuitry formed around REST and TLX (Figures 3A,B).

Both miR-124 and miR-9 are connected to the anti-neural transcription factor REST (Conaco et al., 2006; Visvanathan et al., 2007; Packer et al., 2008; Laneve et al., 2010). While in undifferentiated cells high levels of REST prevent the expression of miR-9 and miR-124, these miRNAs are up-regulated during differentiation and enforce their own expression by targeting *Rest* and its cofactors *CoRest* (*Rcor2*) and *Scp1* (Figure 3A; Conaco et al., 2006; Visvanathan et al., 2007; Packer et al., 2008; Laneve et al., 2010). In the murine dentate gyrus, *Rest* is down-regulated during the transition of aNSCs to neuroblasts before it is up-regulated again in mature hippocampal granule cells (Gao et al., 2011). Adult NSC-specific depletion of *Rest* in the dentate gyrus results in premature differentiation and exhaustion of the aNSC pool (Gao et al., 2011). Of note, *Rest*-depleted aNSCs also show tremendous differences in their miRNA expression profile suggesting that dysregulation of miRNAs may at least in part underlie the imbalance of aNSC maintenance and differentiation induced by *Rest*-deficiency (Gao et al., 2012).

Another important transcription factor interacting with miRNAs to control NSC proliferation and differentiation is the nuclear receptor TLX (*Nr2e1*). TLX is expressed in neurogenic regions during development and adulthood, and its presumed function is to prevent premature differentiation and maintain the undifferentiated self-renewing state of NSCs (reviewed by Islam and Zhang, 2015). Conditional ablation of *Tlx* in dentate gyrus aNSCs does not induce differentiation, but results in precocious cell cycle exit of aNSCs that enter a quiescent inactive state (Shi et al., 2004; Zhang et al., 2008; Niu et al., 2011). Studies performed in mouse primary forebrain aNSCs revealed that miR-9 and let-7 promote neuronal and glial differentiation by targeting *Tlx* (Zhao et al., 2009, 2010). *In utero* electroporation into the lateral ventricles to overexpress miR-9 or let-7 during embryonic development resulted in a similar effect with a decrease of proliferative cells and an increase of differentiating, migrating cells (Zhao et al., 2009, 2010), indicating that miR-9 and let-7 induce differentiation both during developmental and adult neurogenesis. Interestingly, TLX has a direct repressive effect on miR-9 expression forming a negative feedback loop (Figure 3; Zhao et al., 2009). Furthermore, TLX also represses miR-137 by recruiting the histone demethylase LSD1 (*Kdm1a*) to the miR-137 locus. *Lsd1* is highly expressed in mouse aNSCs and declines during neuronal differentiation. Inhibition of LSD1 results in a decrease of aNSC proliferation *in vitro* as well as in the adult dentate gyrus (Sun et al., 2010). Experiments in embryonic NSCs revealed that miR-137 promotes neuronal differentiation by targeting *Lsd1* (Sun et al., 2011), thus adding another loop to the feedback circuity formed around TLX (Figure 3B).

#### MicroRNAs Interacting With Epigenetic Regulators to Balance aNSC Proliferation and Differentiation

Another recurring theme in the context of miRNA-based regulation is the interplay of miRNAs and epigenetic regulators, such as chromatin modifiers (reviewed by Jobe et al., 2012; Lopez-Ramirez and Nicoli, 2014; Murao et al., 2016). miRNA-137, for instance, was reported to target the histone methyltransferase EZH2. EZH2 is part of the polycomb group protein complex involved in epigenetic remodeling by histone methylation. In line with that, overexpression of miR-137 in aNSCs resulted in an overall reduction of H3K27 tri-methylation (Szulwach et al., 2010). The same study also reported an increase of aNSC proliferation at the expense of neuronal differentiation upon miR-137 overexpression (Szulwach et al., 2010), which is opposed to the pro-differentiation function of miR-137 reported in embryonic NSCs (Sun et al., 2011). It was further shown that expression of miR-137 in aNSCs depends on the action of another epigenetic regulator, the DNA methyl-CpG-binding protein MECP2, which in cooperation with SOX2 retains miR-137 expression (Szulwach et al., 2010). Thus, miR-137 may act in concert with several epigenetic regulators (LSD1, MECP2, and EZH2) to control global gene expression (Figures 3B,D). The question is, though, which of these targets are responsible for the distinct effects of miR-137 during proliferation and differentiation of NSCs.

Another example for the interaction of miRNAs with epigenetic regulators is the interaction of the methyl-CpG-binding protein MBD1 with miR-184 and miR-195 in dentate gyrus aNSCs (Figure 3C; Liu et al., 2010, 2013). *Mbd1* is abundantly expressed in the adult brain with highest concentrations in the SGZ of the dentate gyrus. In line with that, *Mbd1*-deficient mice display reduced adult neurogenic activity and impaired hippocampal function (Zhao et al., 2003). Adult NSCs lacking *Mbd1* accumulate at the level of IPCs and fail to transition to the neuronal fate (Jobe et al., 2017). Besides regulating the expression of lineage differentiation-associated protein-coding genes (Jobe et al., 2017), MBD1 was also found to repress the expression of miR-184 and miR-195 by direct binding to their proximal genomic regions (Liu et al., 2010, 2013). Both miR-184 and miR-195 have been shown to promote aNSC proliferation at the expense of neuronal differentiation *in vitro* as well as *in vivo*. *Bona fide* targets identified in this context were *Numbl* for miR-184 (Liu et al., 2010) and *Mbd1* for miR-195, representing yet another example of a negative feedback loop to reinforce miRNA expression (Liu et al., 2013). Additionally, there seems to be some functional redundancy between the two MBD1-regulated miRNAs, miR-195 and miR-184, as the effect induced by miR-195 overexpression could be rescued by miR-184 inhibition (Liu et al., 2013).

### MicroRNAs Regulating Survival in the Adult Neurogenic Niche

An important feature determining the neurogenic output of aNSC is the survival of their neuronal progeny. In fact, under normal conditions, the majority of newborn dentate gyrus neurons undergoes apoptosis within the first days of their life with apoptotic neurons being cleared out by microglia (Sierra et al., 2010; reviewed by Kim and Sun, 2011). Several miRNAs have been shown to regulate survival-associated genes, making them an interesting target for neuroprotective or cell replacement strategies in neurodegenerative diseases (Zhang et al., 2018), stroke (Sun et al., 2017), and epilepsy (Schouten et al., 2015; Yuan et al., 2016; Bielefeld et al., 2017).

A series of studies has been focusing on the impact of global miRNA loss using conditional knockout mouse (cKO) lines for *Dicer*, the key enzyme of miRNA biogenesis (e.g., Davis et al., 2008; De Pietri Tonelli et al., 2008; Hébert et al., 2010; Konopka et al., 2010; Li et al., 2011; Cheng S. et al., 2014). While these studies came to sometimes contradictory results [see Barca-Mayo and De Pietri Tonelli (2014) for a detailed comparison], many of them report that *Dicer* depletion in the embryonic mouse forebrain reduces forebrain growth, increases apoptosis, and leads to premature death of the animals (Davis et al., 2008; De Pietri Tonelli et al., 2008; Hébert et al., 2010; Li et al., 2011). Likewise, specific deletion of *Dicer* in forebrain (cortical and hippocampal) neurons at postnatal stages also resulted in enhanced apoptosis and neuronal loss (Konopka et al., 2010; Cheng S. et al., 2014). Taken together, these findings indicate that a functional miRNA system is critical for neuron survival, but they do not provide direct information on the impact of the miRNA system on the generation and survival of adult-born neurons.

This question was recently assessed by two studies, in which *Dicer* was specifically deleted in aNSCs (Cernilogar et al., 2015; Pons-Espinal et al., 2017). In the first study, *Dicer* was inactivated by delivery of Cre recombinase into primary neural progenitors isolated from the adult SVZ or by retroviral injection into the adult hippocampus of *Dicer*^flox/flox^ mice. Both *in vitro* as well as *in vivo* an increase in doublecortin *Dcx* expression was noted, hinting to an enhanced neuronal differentiation upon *Dicer* deletion (Cernilogar et al., 2015). However, the authors also observed a reduced viability of *Dicer*-depleted cells in their *in vivo* experiments. Pons-Espinal et al. (2017) used the Split-Cre approach developed by Beckervordersandforth et al. (2014) to selectively inactivate *Dicer* in aNSCs in the dentate gyrus and performed experiments on primary aNSCs isolated from *Dicer*^flox/flox^ mice that were nucleofected with Cre recombinase. In contrast to the earlier study by Cernilogar et al. (2015), they observed an impaired neurogenic activity and a bias toward astrocytic differentiation. Furthermore, they noted a reduced survival of *Dicer*-depleted aNSCs both *in vivo* as well as *in vitro*, while the proliferative capacity of *in vitro* cultured aNSCs was not affected. This is in line with previous studies on embryonic NSCs reporting that the ability for NSC self-renewal in culture does not depend on Dicer activity (De Pietri Tonelli et al., 2008; Andersson et al., 2010). These *Dicer* cKO embryonic NSCs were, however, compromised with regard to their differentiation capacity (Andersson et al., 2010). Furthermore an increased cell loss was observed during differentiation of *Dicer*-depleted NSCs (embryonic or adult) indicating that differentiated cells are more sensitive toward global miRNA loss than undifferentiated cells (De Pietri Tonelli et al., 2008; Pons-Espinal et al., 2017).

These two findings prompted the hypothesis that a functional miRNA system is particularly important for cell fate transitions (De Pietri Tonelli et al., 2008; Andersson et al., 2010). Following this notion, Pons-Espinal et al. (2017) focused on a pool of 11 miRNAs (including miR-124 and miR-134) that was found to be highly up-regulated during early neuronal differentiation and asked whether these miRNAs could rescue the bias toward astrocytic differentiation and the impaired neuronal differentiation of *Dicer* cKO aNSCs (Pons-Espinal et al., 2017). Interestingly, they found that only combined delivery of all 11 miRNAs, but not subsets of them, was able to rescue the *Dicer* deletion phenotypes (Pons-Espinal et al., 2017). By combining proteomics and *in silico* miRNA target gene prediction they further found that quite a number of targets are shared by at least 2 of the 11 miRNAs, which led them to speculate that these 11 miRNAs may have a cooperative function targeting an overlapping set of genes to induce neuronal differentiation of aNSC. In fact, cooperative binding of several miRNAs to the same target mRNA is an important feature of the miRNAs’ mode of action and may create functional redundancy (reviewed by Barca-Mayo and De Pietri Tonelli, 2014). However, the data generated by Pons-Espinal et al. (2017) suggest that the level of redundancy among the 11 miRNA is rather low since only the combination of all miRNAs was able to compensate for *Dicer* cKO and not the individual miRNAs.

### MicroRNAs Regulating Migration and Neurite Morphogenesis of Adult-Born Neurons

MicroRNAs also influence functional integration of the neuronal progeny, i.e., migration to their final homing site, neuronal morphogenesis, and synaptogenesis. Immature neuroblasts that arise from the SGZ first have to migrate up into the granule cell layer before they start extending dendrites toward the molecular layer of the dentate gyrus and grow axons that target the CA3 region of the hippocampus, approximately 1 week after their birth (Figure 1C) (Zhao et al., 2006). The maturation process in adult neurogenesis differs from that of embryonic neurogenesis in that adult-born neurons have to integrate into already existing coordinated neuronal networks and that they receive synaptic inputs early on. As shown for mouse hippocampal neurogenesis, immature adult granule cells initially receive excitatory GABAergic inputs, which become inhibitory by 2 weeks after their birth (reviewed by Gonçalves et al., 2016). Around the same time, immature adult-born granule cells also receive glutamatergic input, start developing dendritic spines, and establish efferent and afferent synapses with the local neuron network (reviewed by Deng et al., 2010). Neurons that fail to develop strong synaptic connections will undergo selective apoptosis (Tashiro et al., 2006; and reviewed by Kim and Sun, 2011). Finally, by 8 weeks after their birth, adult-born granule neurons are considered to be fully mature and are indistinguishable from their earlier-born neighbors (Laplagne et al., 2006; and reviewed by Deng et al., 2010).

The molecular mechanisms guiding adult-generated granule cells to their final homing site in the granule cell layer and their mode of migration have not yet been completely resolved. Interestingly, it has been reported that adult-born dentate gyrus neuroblasts first undergo tangential migration along the blood vasculature in the SGZ followed by radial migration to reach the granule cell layer (Sun et al., 2015). While miRNAs have been shown to regulate various genes involved in neuron migration during development (reviewed by Rajman and Schratt, 2017), there is only one study that specifically assessed the impact of miRNAs in the context of adult-born neuron migration (Han et al., 2016). In this study, Han et al. (2016) discovered that elevated levels of miR-19 increased cell migration of *in vitro* cultured hippocampal neural progenitors. They further showed that ectopic expression of miR-19 in the dentate gyrus triggered newborn granule cells to migrate deeper into the granule cell layer. Likewise, neuroblasts generated from the SVZ were found to cover longer distances within the RMS upon miR-19 overexpression. These data indicate that miR-19 promotes migration of adult-born neurons (Han et al., 2016). This function seems to be at least in part mediated by miR-19 targeting *Rapgef2*, which regulates cell migration by modulating the activity of RAP proteins (Han et al., 2016). Knock-down of *Rapgef2* in neural progenitors mimicked the effect of miR-19 overexpression as it promoted migration of *in vitro* cultured neural progenitors and newborn granule cells in the dentate gyrus granule cell layer. Since abnormal migration of adult-born hippocampal neurons was also described in schizophrenia (Duan et al., 2007; Kim et al., 2009), and rare inherited copy number variants of *RAPGEF2* have been associated with familiar schizophrenia (Xu et al., 2009), Han et al. (2016) went on to study the role of miR-19 and *RAPGEF2* in schizophrenia patient-derived hippocampal neural progenitor cells (SZ-NPCs). Indeed, they found that miR-19 is up-regulated in SZ-NPCs and that the regulation of *RAPGEF2* by miR-19 is conserved in humans (Han et al., 2016). miRNA-19 belongs to the family of polycistronic miR-17 clusters, including also miR-17-92 and miR-106b-25b, which have been shown to be important for aNSC proliferation (Brett et al., 2011; Jin et al., 2016). Hence, it would be interesting to assess whether the other members of the miR-17 clusters are also altered in schizophrenia and whether they contribute to the disease.

**FIGURE 4:**
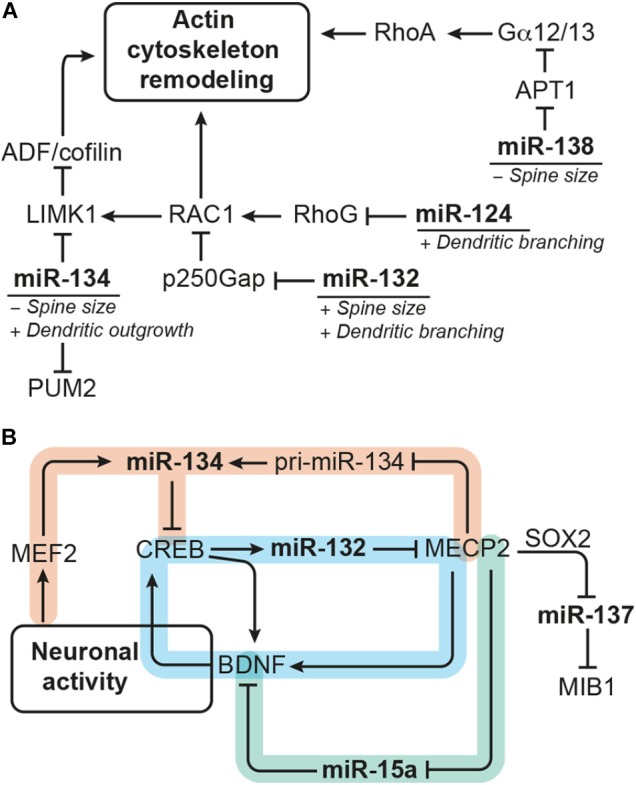
miRNAs modulating dendrite morphogenesis converge by targeting components of the actin remodeling complex and cross talk with each other via neuronal activity-dependent gene regulatory networks. **(A)** miRNA-124, miR-132, miR-134, and miR-138 have divergent effects on dendrite morphogenesis by targeting different components of the actin remodeling pathway. miRNA-124 and miR-134 reduce the activity of RAC1/LIMK1 signaling, whereby miR-124 targets *RhoG* and miR-134 targets *Limk1*. miRNA-132, in contrast, increases RAC1/LIMK1 activity by inhibiting p250Gap-mediated repression of RAC1. miRNA-138 increases Gα12/13 palmitoylation and downstream RhoA signaling by targeting the mRNA coding for the depalmitoylation enzyme APT1. An additional target of miR-134 is the translational repressor PUM2 (Pumilio2) through which miR-134 promotes overall dendritogenesis. **(B)** Many of the miRNAs modulating dendrite morphogenesis engage in regulatory circuits with transcription regulators that respond to neuronal activity. One such regulatory circuit is formed around miR-132 (highlighted in blue), whose expression is induced by CREB downstream of neuronal activity. miRNA-132 itself may influence CREB activation via interfering with MECP2-induced expression of *Bdnf*, which forms a positive feedback loop with CREB. The regulation of miR-134 expression (in orange) seems to be more complex: miR-134 expression was found to be induced by MEF2 downstream of neuronal activity and inhibited by MECP2, which interferes with pri-miR-134 processing. miRNA-134, in turn, targets *Creb* and may thereby provide a negative feedback signal for activity-induced gene expression and also influence miR-132 transcription. Another regulatory hub involves miR-15a (in green), the expression of which is inhibited by MECP2. miRNA-15a targets *Bdnf* mRNA, thereby providing an alternative route through which MECP2 promotes *Bdnf* expression. MECP2 further represses expression of miR-137 by cooperating with SOX2. miRNA-137 inhibits neuronal maturation and dendrite morphogenesis by targeting the ubiquitin ligase MIB1.

After the immature neuroblasts have reached the granule cell layer, they develop into mature neurons, extend dendrites and axons, and establish synaptic contacts. Numerous miRNA-target pairs have been identified to regulate neurite outgrowth and morphogenesis as well as synaptic plasticity (e.g., reviewed by Bicker et al., 2014; Hu and Li, 2017; Rajman and Schratt, 2017). Some miRNAs even show a specific synaptic localization, and several miRNAs are regulated in response to neuronal activity (reviewed by Hu and Li, 2017). Here, we focus on those miRNAs that have been shown to regulate neuronal morphogenesis of hippocampal neurons. Due to the limited number of studies addressing morphogenesis of adult-generated neurons, we also include studies on the role of miRNAs regulating neurite formation and maturation in the developing hippocampus (see Table 2 for an overview of the miRNAs discussed). In the following paragraphs, we highlight examples of miRNAs that converge on functionally related targets or are regulated by the same set of transcription factors (Figure 4).

One common pathway through which miRNAs modulate neuronal morphogenesis is Rho GTPase signaling (Figure 4A). Rho GTPase-mediated remodeling of the actin cytoskeleton is important for regulating several aspects of neuronal morphogenesis (reviewed by Stankiewicz and Linseman, 2014) including dendritic spine maturation of hippocampal neurons (Vadodaria et al., 2013). miRNA-124 promotes axonal and dendrite complexity of rat embryonic hippocampal neurons by inhibiting expression of *Rhog* GTPase (Franke et al., 2012). miRNA-134 negatively affects the size of dendritic spines of *in vitro* cultured embryonic rat hippocampal neurons by targeting *Limk1*, which regulates actin filament dynamics via inhibition of ADF/cofilin (Schratt et al., 2006). In addition, miR-134 was shown to have a growth-promoting effect on dendrites, which is mediated by Pumilio2 (*Pum2*), a translational repressor involved in dendritogenesis (Fiore et al., 2009). miRNA-132 enhances dendrite growth and spine maturation of *in vitro* cultured neonatal rat hippocampal neurons by inhibiting expression of the Rho GTPase activating protein p250Gap (*Arhgap32*), which results in an increased RAC actin remodeling signal and LIMK1 activation (Wayman et al., 2008). Furthermore, miR-138 has been described to negatively regulate the size of dendritic spines by targeting the mRNA coding for the depalmitoylation enzyme APT1 (Siegel et al., 2009). Inhibition of *Apt1* expression by miR-138 leads to an increased palmitolyation and membrane-tethering of G protein Gα12/13, which activates RhoA signaling – another component of the actin remodeling pathway.

MicroRNA expression is modulated by transcriptional regulators, and many of the miRNAs involved in dendrite morphogenesis have been found to be targets of neuronal activity-associated gene regulatory networks. The transcription factors operating in those gene regulatory networks are by themselves often subject to miRNA regulation. Experiments in rat hippocampal neurons showed that expression of the polycistronic miR-379-410 cluster, which also contains miR-134, is induced by the transcription factor MEF2 in response to membrane depolarization or BDNF treatment (Fiore et al., 2009). However, BDNF treatment was also shown to relieve miR-134-mediated repression of *Limk1* in dendrites of embryonic rat hippocampal neurons via a yet unknown mechanism (Schratt et al., 2006). Another miRNA up-regulated in response to neuronal activity is miR-132, the expression of which is induced by the transcription factor CREB. CREB contributes to activity-induced refinement of dendrite morphology and is regulated by miR-134 (Gao et al., 2010). In addition, miR-132 fine-tunes BDNF-mediated CREB activation by targeting *Mecp2*, which induces *Bdnf* (Klein et al., 2007). Recently, it has also been shown that MECP2 can directly interact with the miRNA processing machinery to inhibit the expression of mature miR-134 (Cheng T.-L. et al., 2014). Thus, miR-132 and miR-134 may cross talk in the context of dendrite morphogenesis through CREB and MECP2 (Figure 4B). MECP2 also interacts with SOX2 to retain the expression of miR-137 (Szulwach et al., 2010), which negatively regulates neuronal maturation and dendrite morphogenesis by targeting the mRNA coding for ubiquitin ligase MIB1 (Smrt et al., 2010). MECP2 was further shown to act as a repressor of miR-15a expression, and miR-15a inhibits dendrite maturation of developmental- and adult-born hippocampal neurons by targeting *Bdnf* (Gao et al., 2015). Thus, MECP2-mediated repression of miR-15a represents an indirect route to promote *Bdnf* expression downstream of MECP2 (Figure 4B).

It is important to note that most of the experiments discussed above were performed in primary cultures of rodent embryonic or neonatal hippocampal neurons (Table 2), thus addressing the effect of miRNAs on neurons born in an early developmental phase. However, the same miRNA-target pairs might be also involved in regulating neurite morphogenesis during adult neurogenesis. Depletion of the miR-132 genomic loci in dentate gyrus aNSCs, for instance, was shown to reduce dendrite complexity of neuronal progenies demonstrating that miR-132 has a positive effect on dendrite maturation both during embryonic and adult hippocampal neurogenesis (Magill et al., 2010). In contrast, miR-137 and miR-15a were demonstrated to have a negative effect on dendrite maturation of adult-born dentate granule cells (Smrt et al., 2010; Gao et al., 2015). Another miRNA specifically studied in the context of adult neurogenesis and neuronal maturation is miR-223. Inhibition of miR-223 in dentate gyrus aNSCs by retroviral miRNA sponge injection resulted in an increased dendrite length, while overexpression of miR-223 in human embryonic stem cell-derived neurons had the opposite effect, suggesting that miR-223 also regulates neuronal morphogenesis both during developmental and adult neurogenesis (Harraz et al., 2014).

Taken together, these examples of miRNAs-target pairs that cross talk at different hierarchy levels illustrate once more the complexity and the importance of the miRNA system for adult neurogenesis. Furthermore, many of the miRNAs involved in neuronal morphogenesis discussed above have also been linked to neurological disorders, like Alzheimer’s disease (miR-132, miR-138), Huntington’s disease (miR-132, miR-137, miR-138), psychiatric disorders (miR-132, miR-134, miR-137, miR-138), and epilepsy (miR-134, miR-138) (reviewed by, e.g., Bicker et al., 2014). It is therefore tempting to speculate that dysregulation of miRNAs and altered neuronal morphogenesis may contribute to the disease pathology.

### A Synopsis of the Diverse Roles of MicroRNAs in Adult Neurogenesis

Adult neurogenesis is a multistep process comprising activation of quiescent aNSCs, their differentiation into committed progenitor cells, neuronal survival, migration and functional integration of newborn neurons (Figure 1D). Transitions along these steps are accompanied by dynamic gene expression changes (Llorens-Bobadilla et al., 2015; Shin et al., 2015; Dulken et al., 2017; and reviewed by Beckervordersandforth et al., 2015). As discussed in this review and also in other reports (e.g., reviewed by Lopez-Ramirez and Nicoli, 2014; Murao et al., 2016), miRNAs are an integral part of the gene regulatory networks driving these changes. Although not all interactions discussed here have been experimentally validated in the context of adult neurogenesis, many of the individual factors involved, e.g., miR-124, miR-9, let-7, and miR-137 on the one side and REST, TLX, and LSD1 on the other side, have been shown to play important roles during adult neurogenesis (see Figure 3 and Table 1 for an overview). Thus, the emerging picture is that miRNAs are frequently engaged in feedback loops with transcription factors and epigenetic regulators importantly involved in regulating adult neurogenesis.

By doing so, miRNAs provide an additional layer to control gene expression programs and may help to ensure the robustness of such programs by dampening perturbations and reducing noise (reviewed by Arora et al., 2013; Lopez-Ramirez and Nicoli, 2014; Osella et al., 2014; Murao et al., 2016). However, miRNAs are also involved in remodeling gene expression programs during neural lineage progression (reviewed by Herranz and Cohen, 2010; Peláez and Carthew, 2012). miRNAs may even exert an instructive effect on cell fate as impressively demonstrated by the finding that miR-9/9^∗^ and miR-124 can induce neuronal conversion of fibroblasts (Yoo et al., 2011). Furthermore, global miRNA loss by *Dicer* depletion seems to evoke stronger effects in differentiating cells than in self-renewing NSCs (derived from either embryonic and adult origin), suggesting that cell fate transitions show a particular dependency on miRNA-based regulation (De Pietri Tonelli et al., 2008; Andersson et al., 2010; Pons-Espinal et al., 2017).

However, some of the miRNA functions might be context-dependent. For instance, miR-9 was found to maintain quiescence of aNSCs in the zebrafish brain (Katz et al., 2016), while in mouse aNSCs it promotes neuronal differentiation of aNSCs (Zhao et al., 2009; Kim et al., 2015). These divergent observations might be due to the different experimental model systems employed but may also reflect two different modes of action of miR-9, i.e., cytoplasmic miR-9 promotes neuronal differentiation via canonical targeting of *Tlx* and *Foxo1* (Zhao et al., 2009; Kim et al., 2015), while nuclear miR-9 maintains aNSC quiescence (Katz et al., 2016). Another example of a miRNA eliciting context-dependent effects is miR-137, which was found to promote differentiation of embryonic NSCs (Sun et al., 2010) but inhibits differentiation of adult NSCs (Szulwach et al., 2010).

#### MicroRNAs Converge on Shared Targets to Control Adult Neurogenesis

Many miRNAs have been reported to have rather mild effects on their target genes (Baek et al., 2008; Selbach et al., 2008). Yet, each miRNA might target several hundreds of mRNAs (Lewis et al., 2005; Lim et al., 2005). This “multiplicity” of miRNA targets means that, although the effect of an individual miRNA on a given target might be rather weak, this miRNA might still exert a meaningful biological effect by acting on different genes with overlapping functions (Barca-Mayo and De Pietri Tonelli, 2014; Fischer et al., 2015). Another important feature of miRNA function is “cooperativity,” which describes that most mRNAs possess binding sites for multiple miRNAs (Barca-Mayo and De Pietri Tonelli, 2014; Schmitz et al., 2014; Fischer et al., 2015). Cooperative binding of several miRNAs to the same target mRNA creates functional redundancy and might compensate for the rather mild repression mediated by individual miRNAs on that given target. It has been suggested that by the interplay of multiplicity and cooperativity, miRNAs may have “converging functions” defined as synergic action of a single miRNA or several miRNAs on multiple targets that belong to the same pathway or are exerting redundant functions (Barca-Mayo and De Pietri Tonelli, 2014). For instance, miR-9 and miR-124 have been shown to drive neuronal differentiation of NSCs by converging on *Rest*, *Baf53a*, and components of the Notch signaling cascade as common target genes (reviewed by Stappert et al., 2014). Other examples of miRNAs with convergent functions and mentioned in this review in the context of adult neurogenesis are let-7 and miR-9, which both target *Tlx* (Figure 3B and Table 1; Zhao et al., 2009, 2013), and miR-184 and miR-195, which are both regulated by MBD1 and promote aNSC proliferation (Figure 3C and Table 1; Liu et al., 2010, 2013). Furthermore, several miRNAs (i.e., miR-124, miR-132, miR-134, and miR-138) have been found to be involved in dendrite morphogenesis by targeting components of the actin remodeling pathway as common denominator (Figure 4A and Table 2; Schratt et al., 2006; Wayman et al., 2008; Siegel et al., 2009; Franke et al., 2012). Another mechanism to coordinate miRNA function is to couple the expression of several miRNAs to a common transcription regulator as shown for MECP2, which regulates the expression of various miRNAs involved in dendrite morphogenesis (Figure 4B; Szulwach et al., 2010; Cheng T.-L. et al., 2014; Gao et al., 2015). miRNA cooperativity may be also reflected by the genomic localization of miRNA genes (reviewed by Olive et al., 2015). Many miRNAs are located in polycistronic clusters that encode for members of different miRNA seed families (Figure 2), as is the case for the paralog clusters miR-17-92, miR-160b-25, and miR-106a-363. Interestingly, these clusters encode for miRNAs with distinct functions in adult neurogenesis. For example, miR-25 promotes aNSC proliferation (Brett et al., 2011), whereas miR-19 enhances neuroblast migration (Han et al., 2016; Figure 1D). It would be interesting to assess the extent of co-expression of these miRNAs and their seed family members in the aNSC compartments.

Taken together, these examples illustrate how miRNAs act in concert with gene regulatory networks and also cooperate with each other by targeting functionally related genes. However, most of the reports mentioned above have focused on the action of a single miRNA-target pair. Future studies should also investigate the cooperative function of miRNAs in the context of adult neurogenesis, as it was addressed by Pons-Espinal et al. (2017).

#### MicroRNAs May Contribute to Heterogeneity in the aNSC Niche

Neural progenitors within the adult neurogenic niche are a heterogeneous population that can be distinguished by their cell cycle status (quiescence versus activated cells), by their differentiation potential (neurogenic or gliogenic), and their differentiation stage (aNSCs, IPCs, and neuroblasts) (reviewed by Giachino and Taylor, 2015; Bonaguidi et al., 2016). miRNAs might be importantly involved in conferring aNSC heterogeneity. miRNA-9, for instance, is specifically found in the nucleus of a subset of quiescent aNSCs in the adult brain of zebrafish and mouse (Katz et al., 2016). Disruption of the nuclear localization of miR-9 leads to an increased activation of aNSCs suggesting that this particular expression pattern of miR-9 is crucial for aNSC quiescence (Katz et al., 2016). In addition, miRNAs are involved in biasing embryonic NSCs to either neurogenic or gliogenic differentiation (reviewed by Rajman and Schratt, 2017; Shimazaki and Okano, 2018). It is well perceivable that these miRNAs might have a similar role in aNSCs. In fact, it is not yet clear whether aNSCs are truly multipotent or whether several neural precursor populations with variable differentiation potencies (neurogenic or gliogenic) exist within the NSC niche (reviewed by Bonaguidi et al., 2016), and it might well be that miRNAs could contribute to this heterogenic differentiation potency. Indeed, in their aNSC-specific *Dicer*-knockout model, Pons-Espinal et al. (2017) discovered a shift toward astrogial differentiation at the expense of neuronal differentiation, which could be rescued by combined delivery of 11 miRNAs.

Recent advances regarding single cell tracing and single cell transcriptomics have led to the assignment of specific gene expression profiles to different cell states and further demonstrated the presence of diverse cell states along the process of adult neurogenesis (Llorens-Bobadilla et al., 2015; Shin et al., 2015; Dulken et al., 2017). These analyses revealed that aNSCs do not exist in only two stages (quiescent and activated aNSCs) but instead move through a continuum of different stages during activation (Llorens-Bobadilla et al., 2015; Dulken et al., 2017). It was further shown that aNSC activation is associated with increased protein synthesis (Llorens-Bobadilla et al., 2015; Shin et al., 2015) as well as with vast expression changes in genes associated with energy metabolism, transcriptional regulation, and signaling pathway integration (Shin et al., 2015). Since miRNAs provide an important mechanism to control mRNA–protein output, and many signaling pathway components as well as transcription factors are regulated by miRNAs, it would be interesting to also analyze miRNA profiles at a single cell level and to assess to what extent miRNA expression in the adult neurogenic niche reflects aNSC heterogeneity.

#### MicroRNAs May Contribute to Homeostasis in the aNSC Niche in Healthy and Disease Conditions

Although there is currently a controversial discussion about the extent and role of adult neurogenesis in humans (Boldrini et al., 2018; Sorrells et al., 2018; and reviewed by Kempermann et al., 2018), there remains a strong interest to decipher the molecular mechanisms governing adult neurogenesis and to identify novel tools to gain control over this process. This direction of research is driven by the idea to recruit aNSCs as endogenous cell source replacing the cells lost due to aging, acute lesions, or neurodegenerative diseases (reviewed by Lindvall and Kokaia, 2010). Since miRNAs have been shown to regulate aNSC activation, proliferation, and differentiation and may thereby contribute to the homeostatic regulation in the adult neurogenic niche, they could be envisioned as targets to harness aNSCs for therapeutics approaches. Promising candidates might be miR-9, let-7, miR-106b-25, and miR-17-92, which regulate the balance between quiescence and activation of aNSCs (Nishino et al., 2008; Brett et al., 2011; Jin et al., 2016; Katz et al., 2016). miRNAs might be even involved in conferring plasticity of adult hippocampal neurogenesis in response to environmental signals as shown for miR-17-92 and stress (Jin et al., 2016). Furthermore, miRNAs might also impact on age-dependent decline of adult neurogenesis, and a number of miRNAs have been found to interact with important aNSC regulators that are also associated with aging, including HMGA2, FOXO1, FOXO3, and TLX (Nishino et al., 2008; Zhao et al., 2009, 2010; Brett et al., 2011; Kim et al., 2015). In addition, a functional miRNA system seems to be important to sustain adult neurogenesis and survival within the neurogenic niche (Cernilogar et al., 2015; Pons-Espinal et al., 2017). Finally, numerous miRNAs are dysregulated in pathophysiological conditions associated with dysfunctional hippocampal neurogenesis, such as epilepsy (reviewed by Bielefeld et al., 2017), stroke (reviewed by Khoshnam et al., 2017), and neurodegenerative diseases (reviewed by Qiu et al., 2014). These miRNAs might represent promising targets to not only tackle the primary cause of the disease but to also counteract the disease-induced impairment of adult neurogenesis.

From an evolutionary perspective, it is noteworthy that adult neurogenesis appears to be less pronounced in human compared to rodent brain (e.g., Sanai et al., 2011; Sorrells et al., 2018). The miRNA-target pairs discussed in this review were all identified in rodents, but considering their sequence similarity they should be largely conserved in humans. Nevertheless, there is quite some evolutionary pressure on the miRNA regulome as indicated by the presence of primate-specific miRNAs (Awan et al., 2017), the evolution of miRNA binding sites on mRNA targets (Gardner and Vinther, 2008), and the acquisition of novel factors regulating miRNA-associated processes across evolution (Pratt and Price, 2016). Thus, elucidating the differences in miRNA-based regulation of murine versus human adult neurogenesis might eventually enable the promotion of adult neurogenesis in humans and their exploitation for regenerative purposes.

## Author Contributions

LS, FK, and OB wrote the manuscript and designed the figures.

## Conflict of Interest Statement

OB is a co-founder of and has stock in Life & Brain GmbH. The remaining authors declare that the research was conducted in the absence of any commercial or financial relationships that could be construed as a potential conflict of interest.
